# Targeting the ERβ/Angiopoietin-2/Tie-2 signaling-mediated angiogenesis with the FDA-approved anti-estrogen Faslodex to increase the Sunitinib sensitivity in RCC

**DOI:** 10.1038/s41419-020-2486-0

**Published:** 2020-05-14

**Authors:** Junfei Gu, Yong Zhang, Zhenwei Han, Lei Gao, Jinfeng Cui, Yin Sun, Yuanjie Niu, Bosen You, Chi-Ping Huang, Chawnshang Chang, Xiaolu Wang, Shuyuan Yeh

**Affiliations:** 1 Department of Urology, the Second Hospital of Hebei Medical University, Shijiazhuang, 050000 China; 2 Departments of Urology, Pathology and The Wilmot Cancer Institute, University of Rochester Medical Center, Rochester, NY 14642 USA; 3 Department of Pathology, the Second Hospital of Hebei Medical University, Shijiazhuang, 050000 China; 4 Tianjin Institute of Urology, The Second Hospital of Tianjin Medical University, Tianjin Medical University, Tianjin, 300211 China; 5 Sex Hormone Research Center and Department of Urology, China Medical University, Taichung, 404 Taiwan

**Keywords:** Urological cancer, Renal cell carcinoma

## Abstract

Sunitinib has been used as the main therapy to treat the metastatic clear cell renal cell carcinoma (ccRCC) as it could function via suppressing the tumor growth and angiogenesis. Yet most ccRCC tumors may still regrow due to the development of sunitinib-resistance, and detailed mechanisms remain to be further investigated. The angiopoietin family includes angiopoietin-1 and angiopoietin-2 (ANGPT-1 and -2). It was reported that estradiol regulates expression of ANGPT-1, but not ANGPT-2, through estrogen receptor α (ERα) in an experimental stroke model. To date, there is no finding to link the E2/ER signal on regulating ANGPT-2. Our study is the first to explore (i) how estrogen receptor β (ERβ) can up-regulate ANGPT-2 in RCC cells, and (ii) how ERβ-increased ANGPT-2 can promote the HUVEC tube formation and reduce sunitinib sensitivity. Mechanistic studies revealed that ERβ could function via transcriptional regulation of the cytokine ANGPT-2 in the ccRCC cells. We found the up-regulated ANGPT-2 of RCC cells could then increase the Tie-2 phosphorylation to promote the angiogenesis and increase sunitinib treatment resistance of endothelial cells. In addition to the endothelial cell tube formation and aortic ring assay, preclinical studies with a mouse RCC model also confirmed the finding. Targeting this newly identified ERβ/ANGPT-2/Tie-2 signaling pathway with the FDA-approved anti-estrogen, Faslodex, may help in the development of a novel combined therapy with sunitinib to better suppress the ccRCC progression.

## Introduction

Renal cell carcinoma (RCC) accounts for approximately 2–3% of all malignant diseases in adults and is the third leading cause of death among urological tumors^1,2^. The incidence and mortality of RCC have been rising for the recent decades. There were about 73,820 new cases and more than 14,770 deaths in 2018 in the United States, and the cause of death is usually closely related to metastasis^3^. The partial nephrectomy or radical nephrectomy is considered to be the best treatment for primary clear cell renal cell carcinoma (ccRCCs), but after resection of the primary renal tumor, the recurrence rate is about 20–30%^4^, and the five-year survival rate is still less than 10%^5^.

RCC is considered resistant to radiation therapy and conventional chemotherapy although targeted therapy has produced robust clinical benefits for some patients. Treating the RCC patients with tyrosine kinase inhibitors (TKIs), including axitinib, pazopanib, and sunitinb, resulted in significant prolongation of progression-free survival in patients. Recently, the combination of ipilimumab plus nivolumab, or the combination of axitinib plus avelumab has become a preferred treatment for advanced RCC patients. Although sunitinib is no longer the preferred first line treatment for RCC in US, another TKI, pazopanib, is still used for some metastatic RCC patients. Both sunitinib and pazopanib have similar anti-cancer mechanisms by inhibiting angiogenesis. Overall, the pre-existing and acquired resistance to TKI therapy curtails the utility of this therapy to be combined with other therapies (such as immunotherapy). Thus, understanding the molecular mechanisms for the development of TKI-resistance remains an important question to be addressed.

There are two major types of estrogen receptors (ERs), including ERα and ERβ. The gene for ERβ, also known as ESR2^6,7^, is more extensively expressed in RCC compared to ERα. ERβ may have different functions in different cancers, including inhibiting human breast cancer cell proliferation^8^, promoting kidney cancer^9^, and has been considered as a prognostic predictor in prostate cancer^10^. Also, it was reported that ERβ could increase the vasculogenic mimicry (VM) formation in lung cancer^11^ and promote bladder cancer metastasis via alterations of miR-92a/DAB2IP signals^12^. Results from human clinical data analysis using TCGA database indicated that higher ERβ expressions lead to a shorter overall survival and a lower disease-free survival in RCC^9^^,13,14^. However, whether ERβ signals are involved in responsiveness of TKI therapy remains to be further investigated.

The angiopoietin/Tie-2 signaling pathway plays important roles for the vascular development and function^15^. Tie-2 is a receptor tyrosine kinase specifically expressed in endothelial cells. ANGPT-1 and ANGPT-2 are ligands binding to Tie-2^16,17^. ANGPT-1 can function as a Tie-2 agonist to promote angiogenesis^17^. Wang et al. report that the ANGPT-2 level is elevated in several tumors compared with normal tissues^16^. In certain cases, ANGPT-2 may function as a Tie-2 antagonist^18^. However, some studies showed that under certain conditions, such as the lack of ANGPT-1^19^ or when the concentration of ANGPT-2 is significantly elevated^20^, ANGPT-2 could function as a partial Tie-2 agonist. Supportively, Wu et al. found that combination of the ANGPT-2 blocker and VEGFR2-TKI could improve overall efficacy in treating micro-metastatic disease after RCC resection^21^. Nevertheless, the functions of ANGPT-2 in RCC and whether it is regulated by ERβ to impact the angiogenesis of endothelial cells remain to be further investigated.

Here, we demonstrate that ERβ in ccRCC cells could function through transcriptional regulation of the ANGPT-2 expression to increase the endothelial cell tube formation via a paracrine regulatory mechanism. Targeting this ERβ/ANGPT-2/Tie-2 mediated tube formation with the small molecule, ICI 182,780 (Faslodex), can lead to increasing the endothelial cell sensitivity to the sunitinib treatment for better suppression of ccRCC progression.

## Materials and methods

### Cell lines

All cell lines, 786-O, A498, Caki-1, 293T and HUVEC cells, were purchased from the American Type Culture Collection (ATCC, Manassas, VA). All cell lines were expanded to passage 3, stored in aliquots in liquid nitrogen, and were used for fewer than 4 months after recovery from cryopreservation. All the cells were authenticated by short tandem repeat (STR) DNA profiling, and were tested to be mycoplasma free using the Universal Mycoplasma Detection Kit (ATCC) yearly. Among those RCC cell lines, the 786-O and Caki-1 cell lines have high endogenous ERβ expressions, and were used to test the knockdown effect of ERβ. The A498 cell line has a low endogenous ERβ and was used to test the over-expression of ERβ effect. Those cells were cultured in Dulbecco’s Modified Eagle’s Media (DMEM, Invitrogen, Grand Island, NY) supplemented with 10% fetal bovine serum (FBS), penicillin (25 units/ml), streptomycin (25 g/ml), and 1% L-glutamine, in 5% (v/v) CO_2_ humidified incubator at 37 °C. Prior to treating cells with estrogen, the RCC cells were starved of estrogen contact by culturing in 5% CS-FBS DMEM media for 72 h. The vascular endothelium HUVEC line^22^ was maintained in ECM media containing 10 ng/ml VEGF165 (PeproTech, Rocky Hill, NJ, USA).

### RCC cell lines with lentiviral expression of ER cDNA of shRNA

Lentiviral pWPI-ERβ, pWPI-vec, pLKO-shERβ#1, pLKO-shERβ#2, or pLKO1-vec, together with the psPAX2 packaging plasmid, and pMD2.G envelope plasmid, were transfected into 293T cells using the calcium phosphate transfection method according to the manufacturer’s instructions (Addgene, Cambridge, MA, USA). After 24–36 h co-transfection, the supernatants were collected and incubated with cells for 24 h in the presence of polybrene (2.5 μg/ml). To generate overexpressed ERβ in A-498 cells, A-498 cells were transduced with lentiviral ERβ cDNA. To knock down ERβ, 786-O and Caki-1 cells were transduced with lentiviral shERβ#1, shERβ#2, or vector control. For ERβ knocked-down stable clones, after lentiviral infection, puromycin (2 μg/ml) was used to select stably transduced cells. If not used immediately, the lentiviral soups were frozen in −80 °C for later use.

### RNA extraction, reverse transcription, and real-time quantitative PCR (qPCR) analysis

For RNA extraction, total RNAs were isolated using Trizol reagent (Invitrogen), and then 2 μg RNA was used for reverse transcription using Superscript III transcriptase (Invitrogen). The qPCR was conducted using a Bio-Rad CFX96 system with SYBR green to determine the mRNA expression level of interested genes. Expression levels were normalized to the expression of GAPDH (mRNA) using the 2^−ΔΔ^^Ct^ method. Primers were summarized in the Supplementary Table S1.

### Western blot analysis

Cells were lysed in lysis buffer on ice, proteins (30–50 µg) were separated on 10% SDS/PAGE gel, and then transferred onto PVDF membranes (Millipore, Billerica, MA). Membranes were blocked by 5% Bovine Serum Albumin (Sigma-Aldrich) for 1 h at room temperature and then incubated with proper dilutions of primary antibodies overnight at 4 °C. The following primary antibodies were used at 1:1000 dilution: GAPDH (Santa Cruz, #sc-166574, Paso Robles, CA), ERβ (Abcam, #N2C2, Cambridge, MA), ANGPT-2 (Gene Tex, #GTX100928, Irvine, CA), and Tie-2 (R&D Systems, # AF313-SP). The p-Tie-2 (Y992) (R&D Systems, # AF2720-SP) was used at 1:500 dilution. The next day, anti-rabbit, anti-mouse or anti-goat IgG secondary antibody was used at the concentration of 1:5000 for 1 h at room temperature and then rinsed with TBST 3 times for 5 min. The bands were visualized using an ECL chemiluminescent detection system (Thermo Fisher Scientific, Rochester, NY). GAPDH was used as a loading control. The cell lysis buffer contained 100 mM Tris-HCl (pH 6.8), 2 mM sodium orthovanadate, 5 mM EDTA, 100 mM DTT, 0.5% bromphenol blue, 10% glycerol, 2% SDS, 100 mM 2-mercaptoethanol and 1 mM PMSF.

### Tube formation assay

The RCC cells were seeded on upper chambers of transwell plates with 0.4 μm pore size polycarbonate membrane filters (VWR, #82050-012, Radnor, PA) and the endothelial cells were seeded on lower chambers. After the co-culture, endothelial cells (HUVEC) were seeded in 6-well plates and incubated with the designated treatments for 72 h. For these experiments, the endothelial cells were maintained in mixed media with conditioned media (CM) and fresh media at the ratio of 1:1^22^. After 72 h, matrigel matrix, with reduced growth factor (BD Biosciences, #354230, Bedford, MA), was thawed, gently mixed to homogeneity using cooled pipettes, and was used to coat the wells of 96-well plates. The plates were then incubated for 1 h at 37 °C to allow the matrix solution solidification. The IC50 of sunitinib in the endothelial cells was approximately 0.1 μM^23,24^, and the IC50 of sunitinib in RCC cells was approximately 5 μM^25,26^. We have repeated those IC50 tests on HUVEC and RCC cell lines. Based on the IC50 results and the previous report of using 1 μM sunitinib for their angiogenesis related HUVEC tube formation study^27^, we used 1 μM sunitinib for the present study to test the sunitinib resistance of HUVEC cells. Then, 2 × 10^4^ endothelial cells were resuspended in 100 μl CM with DMSO or 1 μM sunitinib (Sigma, #PZ0012, St Louis, MO), and loaded on top of the matrigel. After incubation at 37 °C for 6–8 h, each well was analyzed under the microscope. The total tube lengths were counted in five randomly microscopic fields (40×) in each well and we used Image J software for quantification. The final results are shown as a relative total tube length or number count (fold). Each sample was run in triplicate and in at least 3 independent experiments (*N* = 3).

### Aortic ring assay

We performed this sunitinib-aortic ring assay following the concentration used in previous article^28^. Another published report^29^ has performed the mouse aortic ring assay with increasing doses of sunitinib (ranging from 0–1000 nM, including 1, 10, 20, 50, 100, 1000 nM). They found that 20 nM was the lowest concentration, that could inhibit the micro-vessel sprouting phenotype of mouse aortic ring. Therefore, we used the concentration of 20 nM to test micro-vessel sprouting under different RCC cell co-culture media. The aortic ring assay was conducted as described^29^. 8-week-old C57BL/6 mice were sacrificed, and their thoracic aortas were procured. We covered a 48-well plates with matrigel matrix with reduced growth factor (70 μl well) and incubated for 30 min at 37 °C under 5% CO_2_. Then, we transferred the aortas into dishes with sterilized 1× PBS buffer. After removing the fibroadipose tissues, we sectioned the aortas into 0.5–1 mm long cross sections, placed those sections on the matrigel-coated wells, and then covered with an additional 70 μl of Matrigel to be entirely embedded in matrigel. Next, 200 μl of CM with or without (w/wo) 20 nM sunitinib were added into wells and incubated at 37 °C under 5% CO_2_. The media was refreshed every 3 days with CM and the indicated drug treatments. On the 9th day, the outgrowth sprout numbers of micro-vessel were imaged and counted in five randomly chosen microscopic fields (40×) in each well and we used Image Pro Plus software for quantification. The final results are shown as a relative total sprout number count. Each sample was run in triplicate and in multiple sets of experiments.

### Chromatin immunoprecipitation assay (ChIP)

Briefly, after 786-O cells were treated with 4% formaldehyde for 10 min to cross-link the protein-genomic DNA complex, they were washed twice with cold 1× PBS, collected, and lysed in SDS lysis buffer containing 1× protease inhibitor cocktail. The lysates were collected and sonicated with a pre-determined power to yield genomic DNA fragments of 300–1000 bp long. Lysates were pre-incubated with protein A-agarose conjugated normal rabbit IgG (sc-2027, Santa Cruz Biotechnology) to reduce non-specific binging background. Anti-ERβ antibody (Abcam, #N2C2, 5 μg) was then added to the cell lysates at 4 °C overnight. Salmon Sperm DNA/Protein A Agarose-50% Slurry was added and incubated for 1 h at 4 °C to collect the antibody/protein/DNA complex. IgG was used as the negative control. Specific primer sets were designed to amplify a target sequence within the human ANGPT-2 gene’s promoter. PCR products were analyzed by agarose gel electrophoresis and qRT-PCR.

### Luciferase reporter assays

The human 5′-promoter region of the ANGPT-2 gene was constructed into pGL3-basic luciferase reporter vector (Promega). Site-directed mutagenesis of the ERβ binding site (ERE) in the 5′ promoter of ANGPT-2 was achieved with the Quick Change mutagenesis. According to the manufacturer’s instructions, the 786-O and A498 cells were plated in 24-well plates and the plasmids were transfected with Lipofectamine 3000 transfection reagent (Invitrogen, Carlsbad, CA) and pRL-TK was used as an internal control, in order to take this value as the baseline control. After transfection for 36–48 h, the luciferase activity was measured by Dual-Luciferase Assay (Promega) according to the manufacturer’s method. Site-directed mutation of the ERβ binding site was designed according to the Quick Change Primer Design Protocol.

### In vivo mouse RCC model and drug administration

For the power analysis, we expect the proportions of drug positivity in treated group vs. the control group are 85 and 10%, a sample size of 8 in each group achieves 85% to detect the proposed difference (based on Fisher’s exact test). Based on the above prediction, we therefore choose 8 mice per group for the designed study. If necessary, we will increase the mouse number to reach statistical significance. Thirty-two 6–8 weeks old athymic NCr-nu/nu female mice were purchased from NCI and received orthotopic implantation of 1×10^6^ 786-O-Luc cells mixed 1:1 with matrigel under the left renal capsule. After 14 days of tumor development, mice were randomly divided into 4 groups and given the following treatments: (1) DMSO control, (2) ICI 182,780 at 2.5 mg/kg, (3) sunitinib at 20 mg/kg, or (4) ICI 182,780 + sunitinib, every other day by intraperitoneal injection. Tumor development and metastasis were monitored by Fluorescent Imager (IVIS Spectrum, Caliper Life Sciences, Hopkinton, MA) once a week. After 4 weeks of treatment, the mice were sacrificed, and tumors and any metastases were removed for studies. The body weights were not significantly changed in mock or ICI182,780 single treatment group. The sunitinib treated mouse groups had reduced body weights, yet they still maintained a reasonable appetite and daily activities. No experimental animals were excluded from data (*n* = 8 for each group). Tumor weights and lengths were measured and calculated by the RCC tumors grown on the left kidney. Two technicians were blinded to the group allocation when assessing the outcome of tumor weights and lengths. The Institutional Animal Care and Use Committee (IACUC) review board at University of Rochester Medical Center approved the studies.

### Immunohistochemical (IHC) staining

Tissues were fixed in 10% (v/v) formaldehyde in PBS, embedded in paraffin, cut into 5 μm sections, dewaxed in a 65 °C incubator for 30 min and then dewaxed in xylene and hydrated in ethanol. For antigen retrieval, the tissues/slides were then placed in sodium citrate (pH = 6), and microwave heated for 30 min. 3% H_2_O_2_-methanol was used to inactivate endogenous peroxidase. After blocking for 30 min in goat serum, tissues were incubated with 1:100 dilutions of indicated antibodies for ERβ (Abcam, #ab288, Cambridge, MA), ANGPT-2 (Proteintech, #24613-1-AP, Rosemont, IL), CD 31 (ABclonal, #A3181, Woburn, MA), or CD 34 (ABclonal, #A13929) overnight at 4 °C. After rinsing with Tris-buffered saline, the slides were incubated for 1 h with biotin-conjugated secondary antibody (Vector Laboratories, Burlingame, CA), and then incubated with horseradish peroxidase (HRP)-conjugated streptavidin. Freshly prepared DAB kit (Vector Laboratories) was used as substrate to detect HRP. Tissue slides were then counter-stained with hematoxylin and mounted with aqueous mounting media.

### Quantification of IHC result

IHC score/quantification was conducted as described^30,31^ Staining was examined at a high-power field (400×) by two experienced pathologists. The results obtained from five random fields were averaged. Average optical density of staining was calculated using Image J software. Each field was judged by a scoring system combining intensity and percentage. In each field, staining intensity was divided into four scores: 0, no staining; 1, weakly positive staining; 2, moderately positive staining; 3, strongly positive staining. According to the staining percentage of the stained cell number, it was graded as: 0 for 0%, 1 for ≤25%, 2 for 25–50%, 3 for 50–75% and 4 for ≥75%. The total score of each section was calculated by multiplying the intensity and percentage scores. After quantification by the Image J software, the data were normalized to the vector control group and the final results are shown as a relative staining intensity (fold).

### Statistics analysis

All experiments were performed in triplicate per each experiment and at least 3 independent times. The experimental results and variance were calculated and shown as the mean ± SD. The variance was similar between the groups in all experiments, and has been statistically compared. Differences in different groups were analyzed by Student’s *t*-test or one-way ANOVA. Statistical analyses involved paired t-test with SPSS Version 22.0 (IBM Corp, Armonk, NY) or GraphPad Prism 6 (GraphPad Software, Inc, La Jolla, CA). *p* < 0.05 was considered statistically significant.

## Results

### ERβ can function via impacting the endothelial cell tube formation to alter the sunitinib sensitivity in RCC

**Fig. 1:**
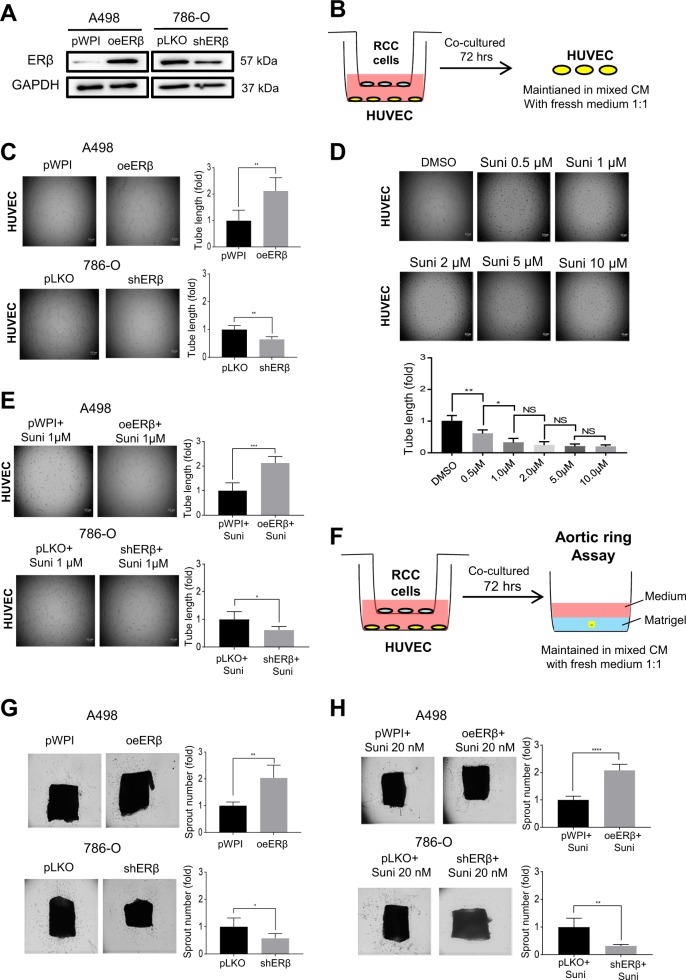
ERβ can function via impacting the endothelial cell tube formation to alter the sunitinib sensitivity in RCC. **a** Western blot verification of ERβ overexpression in A498 cells (oeERβ, left) and knockdown in 786-O cells (shERβ, right). **b** Outline of co-culture system. Endothelial cells (HUVEC) were co-cultured with ccRCC cells for 72 h, and then the endothelial cells were harvested. After digesting cells, the endothelial cells were maintained in 1:1 mixed conditioned media (CM) from co-culture and fresh media. **c** We show that overexpressing ERβ (oeERβ) in A498 cells increased HUVEC tube formation and knocking down ERβ (shERβ) in 786-O cells decreased HUVEC tube formation. **d** We found sunitinib could suppress HUVEC cell tube formation in a dose dependent manner, from DMSO, 0.5, 1, 2, 5, to 10 μM. **e** The results revealed that oeERβ increased the HUVEC resistance to 1 μM sunitinib treatment, and shERβ decreased the HUVEC resistance to 1 μM sunitinib treatment. **f** Outline of aortic ring assay. Aortic rings were embedded in the matrigel, then maintained in 1:1 ratio of mixed CM from co-culture system and fresh media. **g** The aortic ring assay to confirm the above results, showing that oeERβ in A498 cells resulted in higher numbers and lengths of microvessels sprouted from the aortic ring, while shERβ in 786-O cells led to suppress the microvessel sprouting from the aortic ring. **h** The aortic ring assay to confirm the sunitinib results, showing that oeERβ in A498 cells and the CM with 20 nM sunitinib resulted in higher numbers and lengths of microvessels sprouting from the aortic ring, while shERβ in 786-O cells and the CM with 20 nM sunitinib led to suppressing the microvessel sprouting from the aortic ring. For **c**, **e**, **g** and **h**, quantitation is at the right and for **d**, quantitation is below the images. Data are presented as mean ± SD, *p < 0.05; **p < 0.01; ***p < 0.001; ****p < 0.0001 compared with the controls, and Scale bar: 5 μm.

Sunitinib malate has been used as the standard medicine to suppress the metastatic ccRCC, although many of these treatments may eventually fail due to development of sunitinib-resistance^32^. Early studies^23^ indicated that sunitinib might suppress the ccRCC growth primarily through an antiangiogenic mechanism without directly targeting the ccRCC cells growth. Other studies also found that ERβ might function via altering the circATP2B1/miR-204-3p/FN1 signaling to increase the ccRCC cell invasion^9^. We first established RCC cell lines with high or low ERβ level by overexpressing ERβ (oeERβ) in ccRCC A498 cells that have a lower endogenous ERβ expression, as well as by suppressing ERβ using shRNA (shERβ) in ccRCC 786-O cells that have a higher endogenous ERβ expression (Fig. 1a).

We then studied the ERβ impacts on the endothelial cell tube formation, a key step to impact the ccRCC metastasis^33^. The CM from 72 h co-culture were collected and mixed with fresh media at 1:1 ratio to assay the HUVEC tube formation (see detailed outline in Fig. 1b).

The results revealed that overexpressing ERβ in A498 cells (A498-oeERβ) increased the endothelial HUVEC tube formation, and suppressing ERβ in 786-O cells (786-O-shERβ) decreased the HUVEC tube formation (Fig. 1c).

Early studies indicated that sunitinib, a popularly used TKI therapy, might inhibit the ccRCC metastasis via suppressing the endothelial cell tube formation^34^, therefore we were interested in seeing if ERβ-altered endothelial cell tube formation may result in altering the sunitinib sensitivity to suppress the ccRCC cell invasion. We first found sunitinib could suppress HUVEC tube formation in a dose dependent manner, from DMSO, 0.5, 1, 2, 5, to 10 μM (Fig. 1d). We then set the sunitinib concentration at 1 μM to study ERβ impact, and results revealed that increasing ERβ via adding ERβ-cDNA increased the HUVEC resistance to the sunitinib treatment, and decreasing ERβ via ERβ-shRNA decreased the HUVEC resistance to the sunitinib treatment (Fig. 1e).

We also applied a different approach using an aortic ring assay (detailed outline in Fig. 1f) to confirm the above results, and results in Fig. 1g, h, revealed that increasing ERβ in A498 cells resulted in higher numbers and lengths of micro-vessels (g) that sprouted from the aortic ring and micro-vessel sensitivity (h) to Sunitinib treatment, while suppressing ERβ in 786-O cells reduced the numbers of the micro-vessel sprouting from the aortic ring (g) and micro-vessel sensitivity (h) to Sunitinib treatment.

Together, results from Fig. 1a–h suggest that ERβ can function via impacting the endothelial cell tube formation to alter the sunitinib sensitivity to suppress the RCC progression.

### Human clinical data analysis showing a higher ERβ expression is associated with poorer prognosis in RCC patients

**Fig. 2:**
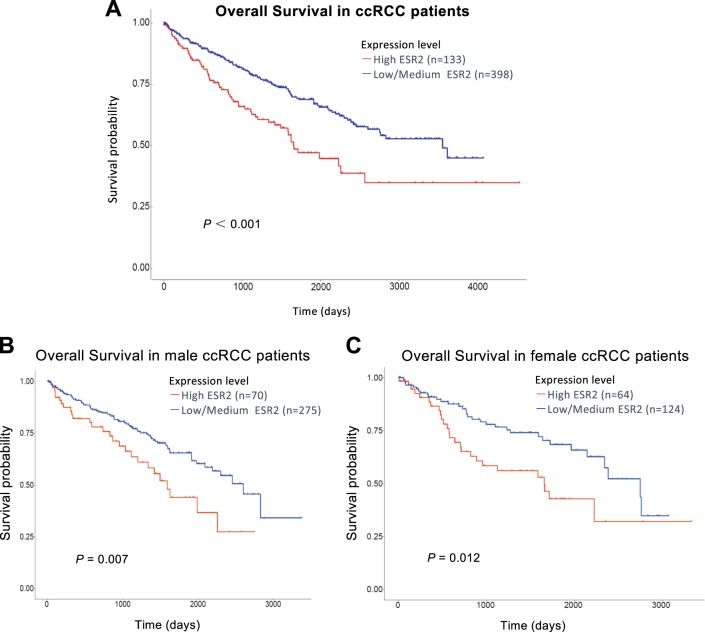
Human clinical data survey showing higher ERβ expression is associated with the poor prognosis in RCC patients. **a** Overall survival curve indicated higher levels of ERβ were associated with a worse survival rate (*p* < 0.001). **b** Overall survival curves indicated that male RCC patients with higher levels of ERβ had shorter survival rate (*p* = 0.007). **c** Overall survival curves indicated that female RCC patients with higher levels of ERβ had a lower survival rate (*p* < 0.012).

To link the above in vitro cell studies to the human clinical studies, we analyzed the clinical significance of ERβ in RCC samples through the UALCAN (http://ualcan.path.uab.edu/) database. The clinical data from TCGA samples showed that patients with higher ERβ (also known as ESR2) mRNA expression had a significantly worse overall survival (*p*<0.001, Fig. 2a), suggesting ERβ might play a positive role to promote the RCC progression.

We further analyzed the survival of different genders, and results indicated that higher expression of ERβ could promote the RCC progression resulting in lower survival rates in male as well as in female RCC patients than low/medium expression ERβ (*p* = 0.007, Fig. 2b, and *p* = 0.012, Fig. 2c, respectively).

Together, results from human clinical sample analysis suggest that a higher ERβ expression was associated with the poor prognosis in ccRCC patients (Fig. 2a–c), which is in agreement with the above in vitro data from multiple ccRCC cells showing higher ERβ may function via up-regulating the HUVEC tube formation to alter the sunitinib sensitivity to promote the ccRCC progression.

**Fig. 3:**
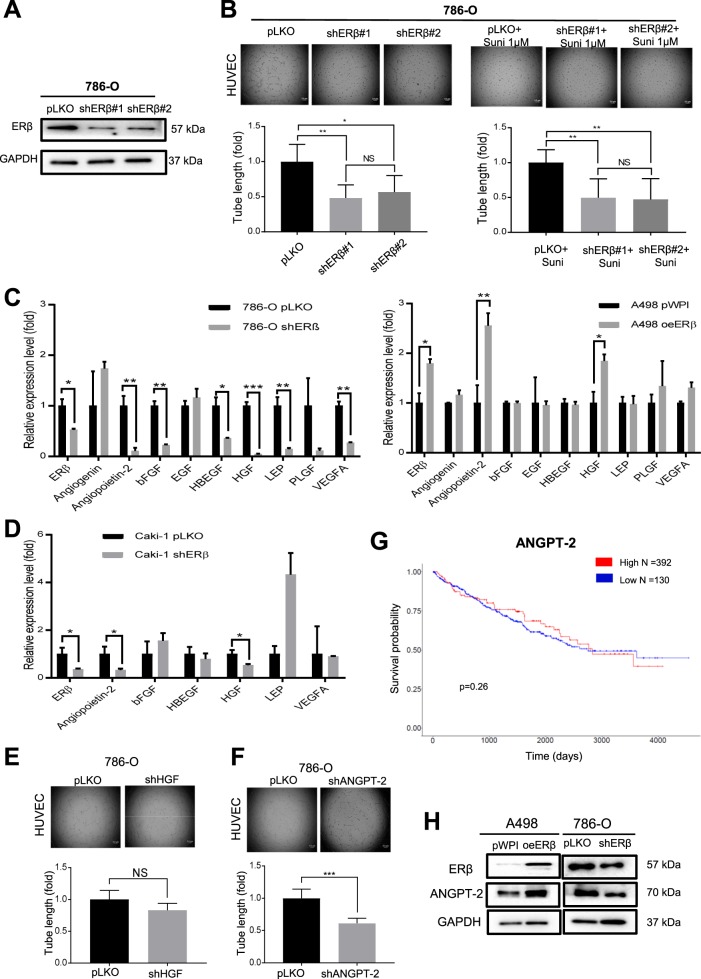
Mechanism dissection of how ERβ can increase the HUVEC tube formation: via increasing the ANGPT-2 expression. **a** Western blot validation of ERβ knockdown by shERβ#1 and shERβ#2 in 786-O cells. **b** The results revealed that shERβ#1 and shERβ#2 in 786-O cells decreased the HUVEC tube formation, and 1 μM sunitinib decreased the HUVEC resistance to sunitinib. **c** The qRT-PCR assay for screening RCC angiogenesis-associated genes in A498 cells with oeERβ and in 786-O cells with shERβ. **d** The qRT-PCR assay (left panels) for screening RCC angiogenesis-associated genes in Caki-1 cells with shERβ. **e** Data showing that knockdown of HGF (shHGF) in 786-O cells did not change the tube formation of HUVEC cells. **f** Knockdown of ANGPT-2 (shANGPT-2) in 786-O cells could decrease HUVEC tube formation. **g** Higher expression levels of ANGPT-2 were associated with poorer prognosis in RCC patients, although there is not statistical significance (*p* = 0.26). **h** The western blot results showed oeERβ in A498 cells increased the Angiopoietin-2 protein expression and shERβ decreased the Angiopoietin-2 protein expression in 786-O cells. For **b**, **e**, and **f**, quantitation is at the right of the images. Data are presented as mean ± SD, *p < 0.05; **p < 0.01; ***p < 0.001, NS = Not Significant, compared with the controls, and Scale bar: 5 μm.

To eliminate ERβ-shRNA off-target effect, we then applied the 2nd ER-shRNA (shERβ#2)^9^ to confirm the ERβ knockdown effects in 786-O cells. Western blot data showed that both shERβ#1 and shERβ#2 could effectively suppress the ERβ expression in 786-O cells (Fig. 3a), and such suppression in 786-O cells (786-O-shERβ) could then lead to decreasing the HUVEC tube formation.

We then found that decreasing the expression of ERβ via ERβ-shRNA#1 or #2 could make the HUVEC more sensitive to the sunitinib treatment (Fig. 3b). Since shERβ#1 has a stronger knockdown effect, we decided to use this in most of the following experiments.

### Mechanism dissection of how ERβ can increase the HUVEC tube formation: via increasing the ANGPT-2 expression

Next, to dissect the mechanism of how ERβ can increase the HUVEC tube formation, we focused on tumor angiogenesis-related genes, including Angiogenin, ANGPT-2, bFGF, EGF, HB-EGF, HGF, Leptin, PLGF, and VEGF-A^35^. We first examined the impacts of altered ERβ expression on their differential mRNA expressions in both RCC (A498 and 786-O) cell lines. Results revealed that altering the ERβ expression led to significantly changing the expression of ANGPT-2 and HGF, overexpression of ERβ in A498 cells can increased their expressions, and knockdown of ERβ in 786-O cells could down-regulate the expressions of ANGPT-2, bFGF, HB-EGF, HGF, Leptin, and VEGF-A (Fig. 3c).

The results from the 3rd RCC cell line, Caki-1 also revealed that knockdown of ERβ led to suppressing the expressions of ANGPT-2 and HGF (Fig. 3d). Similar results were also obtained when we replaced qPCR mRNA assay in the Caki-1 cells (Fig. 3d). We then tested the impacts of knocking down HGF (plko-shHGF) and ANGPT-2 (plko-shANGPT-2) in 786–0 cell to further validate their roles on the HUVEC tube formation. Results revealed that only plko-shANGPT-2, but not the plko-shHGF, could decrease the HUVEC tube formation (Fig. 3e, f).

Importantly, results from human clinical sample surveys using TCGA database and Oncolnc (http://www.oncolnc.org/) also revealed that the RCC patients with higher ANGPT-2 expression had a poor overall survival, although there is no statistical significance (*p* = 0.26, Fig. 3g). Western blot data also showed that adding ERβ in A498 cells led to increasing the ANGPT-2 protein expression in the A498 cells and knocking down ERβ in 786-O cells decreased the ANGPT-2 protein expression in the 786-O cells (Fig. 3h).

Together, results from Fig. 3a–h suggest that ERβ can promote the HUVEC tube formation via increasing the ANGPT-2 expression.

### ERβ can increase the HUVEC tube formation via altering the ANGPT-2/Tie-2 axis

To further dissect the mechanism of how ERβ-increased ANGPT-2 can increase the HUVEC tube formation, we focused on the Tie-2, as recent studies indicated that Tie-2 is primarily expressed on endothelial cells. Coffelt et al also found that ANGPT-2 could function as a Tie-2 agonist on human Tie-2^+^ monocytes to enhance their pro-angiogenic activity in vitro^36^.

**Fig. 4:**
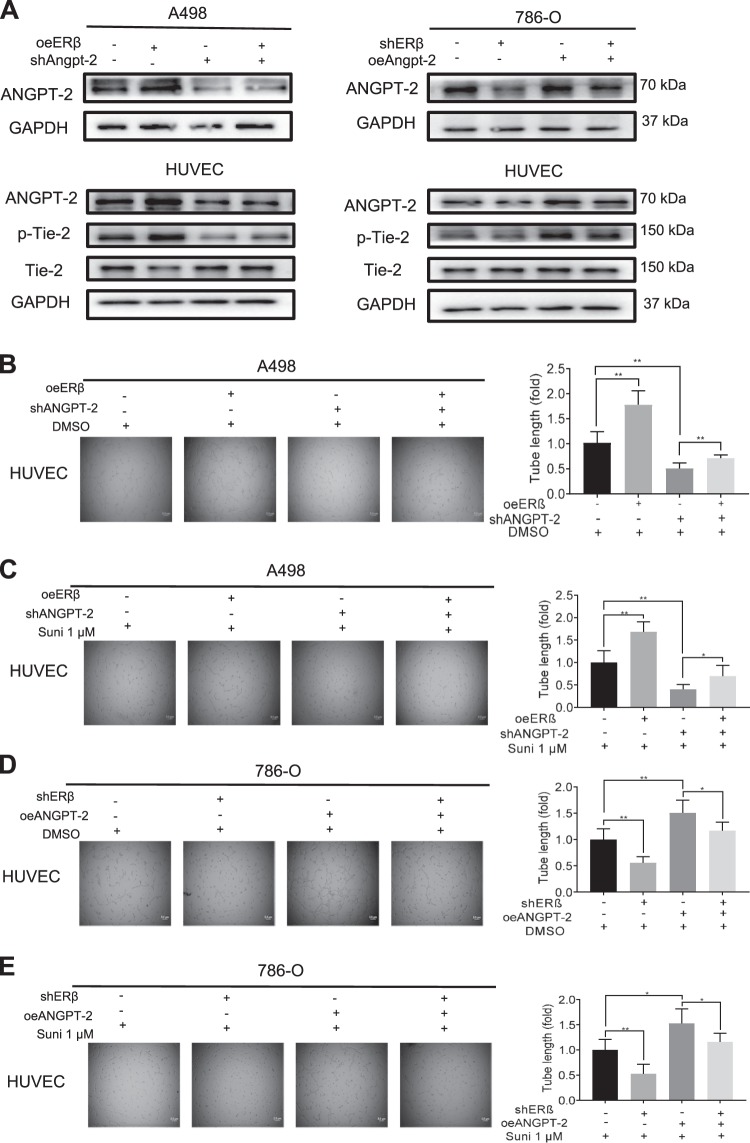
ERβ signals could increase Angiopoietin-2 and up-regulate the Angiopoietin-2/Tie-2 axis. **a** Protein expressions of ANGPT-2 of RCC cells and ANGPT-2, Tie-2 and p-Tie-2 of HUVEC cells. Western blot assays were performed on A498 cells transfected with pWPI+pLKO, oeERβ + pLKO, pWPI+shANGPT-2, oeERβ + shANGPT-2 (left panels), and on 786-O cells transfected with pLKO+pWPI, shERβ + pWPI, pLKO+oeANGPT-2, shERβ + oeANGPT-2 (right panels). **b**–**e** Tube formation assays were performed using (**b**) A498 cells transfected with pWPI+pLKO, oeERβ + pLKO, pWPI+shANGPT-2, oeERβ + shANGPT-2, using (**c**) A498 cells transfected with pWPI+pLKO, oeERβ + pLKO, pWPI+shANGPT-2, oeERβ + shANGPT-2, and treated with1 μM Sunitinib, using (**d**) 786-O cells transfected with pLKO+pWPI, shERβ + pWPI, pLKO+oeANGPT-2, shERβ + oeANGPT-2 and (**e**) using 786-O cells transfected with pLKO+pWPI, shERβ + pWPI, pLKO+oeANGPT-2, shERβ + oeANGPT-2, and treated with1 μM sunitinib. For **b**–**e**, quantitation is at the right of the images. Data are presented as mean ± SD, *p < 0.05; **p < 0.01, compared with the controls, and Scale bar: 5 μm.

To study if the RCC cells secreted ANGPT-2 may affect the phosphorylation of Tie-2 on endothelial cells, we constructed and infected the pLKO-shANGPT-2 and pWPI-oeANGPT-2 plasmids in the RCC cells. The results from interruption assays using sh-ANGPT-2 revealed that suppressing ANGPT-2 expression led to partly reversing the oeERβ-increased ANGPT-2 expression in RCC A498 cells. As expected, suppressing ANGPT-2 expression also led to decrease the phosphorylation of Tie-2 on endothelial cells (Fig. 4a, left panels), and oeERβ could increase angiogenesis using tube formation assay (Fig. 4b), as well as the resistance to the sunitinib treatment of HUVEC cells (Fig. 4c).

In contrast, results from an interruption assay using oe-ANGPT-2 can then lead to partly reversing the shERβ-decreased ANGPT-2 expression in 786-O cells, which might then increase the ANGPT-2 expression and phosphorylation of Tie-2 on the endothelial cells (Fig. 4a, right panels), and shERβ-decreased angiogenesis using in HUVEC cell tube formation (Fig. 4d), and shERβ-decreased the HUVEC resistance to the sunitinib treatment (Fig. 4e).

Together, results from Fig. 4a–e suggest that ERβ can promote the HUVEC cell tube formation and increase the HUVEC cell resistance to the sunitinib treatment via increasing the ANGPT-2/Tie-2 signaling.

### ERβ can increase ANGPT-2 expression via transcriptional regulation

**Fig. 5:**
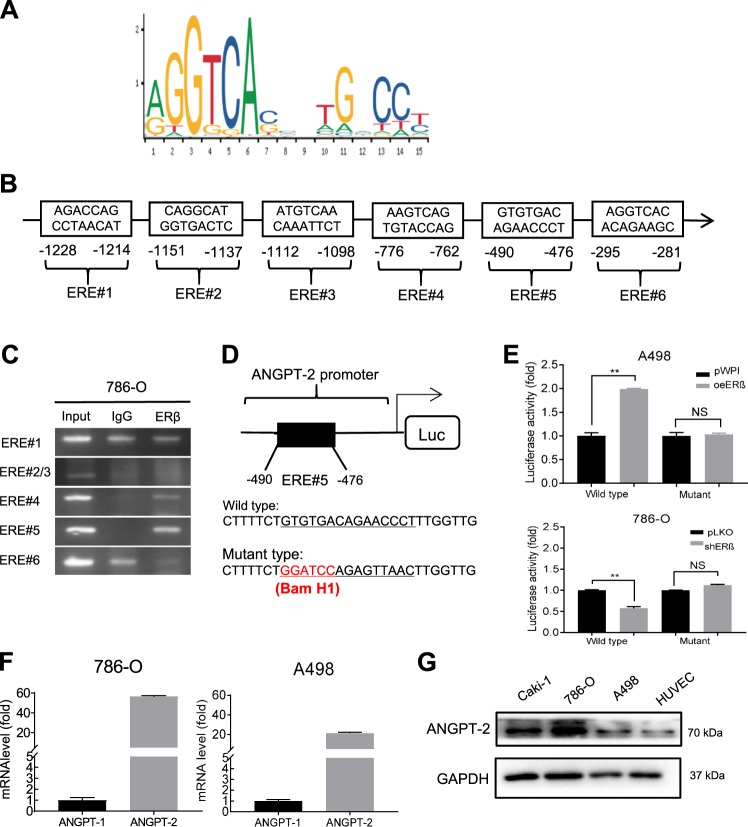
Mechanism dissection of how ERβ can increase Angiopoietin-2: via promoter ERE binding and transcriptional regulation. **a** Putative ERE motif locations on ANGPT-2 promoter and sequences are shown. **b** Six putative EREs on the ANGPT-2 promoter as predicted by JASPAR. **c** ChIP assay results of the ANGPT-2 promoter EREs in 786-O cells. **d** Diagram (upper panel) of cloning the 2 kb ANGPT-2 promoter into pGL3 basic luciferase report vector (pGL3). Site-directed mutagenesis (lower panel) of ERE#5 by mutating part of the ERE#5 sequence into BamH1 site (-GGATCCAGAGTTAAC-). **e** Co-transfection of pGL3-Luciferase constructs with ERE#5 wildtype or mutant ANGPT-2 promoter into, A498 cells with/without oeERβ (upper) and into 786-O cells (lower) with/without shERβ. The luciferase assay was applied to detect the promoter activity. **f** Detection of the relative mRNA expressions of ANGPT-1 and ANGPT-2 in A498 (upper) and 786-O (lower) cells. **g** Western blot assays were performed on Caki-1, 786-O, A498 and HUVEC, to detect the protein of ANGPT-2. Data are presented as mean ± SD, **p < 0.01; NS = not significant.

To dissect the mechanism of how ERβ can increase the ANGPT-2 expression, we first noticed that ERβ could increase the expression of ANGPT-2 at the mRNA (Fig. 3c) and protein levels (See Fig. 3h). We then studied whether ERβ can increase the expression of ANGPT-2 at the transcriptional level. We first applied the JASPER database to search for potential ERβ response elements (EREs) on the 2 kb promoter region of ANGPT-2 (Fig. 5a), and found six putative EREs (−1228 to −1214; −1151 to −1137; −1112 to −1098; −776 to −762; −490 to −476; −295 to −281) (Fig. 5b). We then applied the chromatin immunoprecipitation (ChIP) assay, and results revealed that ERβ could bind to the ERE#5 located at the −490 to −476 bp from the transcriptional start site of ANGPT-2 (Fig. 5c).

We then mutated the key sequences of this ERE#5 (Fig. 5d), and applied the luciferase reporter assay. The results revealed that over-expressing ERβ in the A498 cells led to increasing the wild type, but not mutated, ERE-luciferase reporter activity (Fig. 5e). As expected, knockdown of ERβ could decrease the luciferase reporter activity in 786-O cells with the wild type ERE, but not mutated ERE (Fig. 5e). We found the mRNA of ANGPT-2 is much higher than ANGPT-1 (Fig. 5f), and the protein expression of ANGPT-2 in ccRCC cells was also higher than that of endothelial cells (Fig. 5g).

Together, results from Fig. 5a–g suggest that ERβ can increase ANGPT-2 expression via transcriptional regulation with direct binding to the ERE (−490 to −476 bp) on the 5′ promoter of ANGPT-2 in RCC cells. The increased ANGPT-2 may then function as an agonist to bind and phosphorylate the Tie-2 receptor on the endothelial cells to promote the angiogenesis.

### Targeting the ERβ/ANGPT-2/Tie-2 signaling with the ERβ antagonist ICI 182,780/Faslodex, can increase the sunitinib sensitivity via inhibiting the endothelial cell tube formation

The ERβ was positively expressed in the RCC cell lines, however, little ERα was detected in the RCC cell lines^13^^,14^. Previous studies indicated that 10 nM E2 could increase ERβ downstream gene expression, which was blocked by adding 1 μM anti-estrogen ICI 182,780 (Faslodex)^13^.

**Fig. 6:**
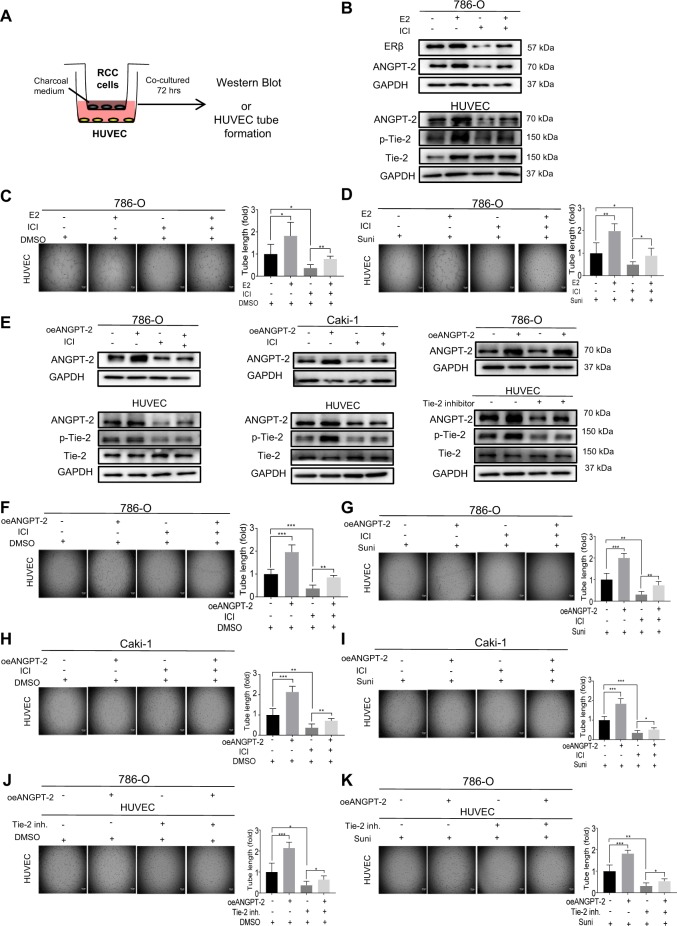
Targeting the ERβ-Angiopoietin-2/Tie-2 signaling with the small molecule ERβ antagonist ICI 182,780 (ICI, Faslodex) can increase the sunitinib sensitivity via inhibiting the endothelial cell tube formation. **a** Outline of co-culture system. The same method as the previous co-culture method. E2 or ICI were added into RCC cells cultural with CS-FBS medium. **b** Detection of protein expression of ANGPT-2 in RCC cells, and the ANGPT-2, Tie-2 and p-Tie-2 in HUVEC cells. Western blot assays were performed on 786-O cells treated with control, 10 nM E2, 1 μM ICI 182,780 and 10 nM E2 + 1 μM ICI. **c**, **d** Tube formation was performed using 786-O cells treated as the above with/without 1 μM sunitinib treatment. **e** To test ANGPT-2 in RCC cells and ANGPT-2, Tie-2 and p-Tie-2 in HUVEC cells, western blot assays were performed on 786-O cells (left panel) transfected with control, oeANGPT-2, or oeANGPT-2+ treated with/without ICI, on Caki-1 cells (middle panel) transfected with control, oeANGPT-2, or oeANGPT-2+ treated with/without ICI. To test ANGPT-2 in RCC cells and ANGPT-2, Tie-2 and p-Tie-2 in HUVEC cells, western blot assays were performed on 786-O cells transfected with control, with or without oeANGPT-2, and HUVEC cells treated with control, with or without Tie-2 inhibitor (right panels). **f**, **g** Tube formation assays were performed using 786-O cells (**f**) transfected with vector control, oeANGPT-2 or oeANGPT-2+ treated with/without ICI, and (**g**) with/without sunitinib. **h**, **i** Tube formation were performed using Caki-1 cells (**h**) transfected with vector control, oeANGPT-2 or oeANGPT-2+ treated with/without ICI, and (**i**) with/without 1 μM sunitinib. **j**, **k** Tube formation were performed using 786-O cells (**j**) transfected with control, oeANGPT-2, and HUVEC treated with control, Tie-2 inhibitor, and (**k**) with/without sunitinib. For **c**, **d**, and **f**–**k** quantitation is at the right of the images. Data are presented as mean ± SD, *p<0.05; **p < 0.01; ***p < 0.001, compared with the controls, and Scale bar: 5 μm.

Results from our co-culture of RCC cells with endothelial cells (detailed outline in Fig. 6a) revealed that treating with 10 nM E2 could increase ANGPT-2 expression significantly in the 786-O and HUVEC cells and the phosphorylation of Tie-2 in the HUVEC cells. Treating cells with 1 μM ICI 182,780 could decrease the ANGPT-2 expression significantly in the 786-O and HUVEC and phosphorylation of Tie-2 in the endothelial cells, while the increase of ANGPT-2 with E2 was blocked by treatment with ERβ selective anti-estrogen ICI 182,780 (Fig. 6b) in both types of cells.

Importantly, results from endothelial tube formation also showed that E2-induced angiogenesis and the E2-increased HUVEC resistance of the sunitinib treatment could be suppressed by treating with the anti-estrogen, ICI182,780 (Fig. 6c, d).

As expected, results from interruption assays also found that oeANGPT-2 increased ANGPT-2 expression in the 786-O and Caki-1 cells and phosphorylation of Tie-2 on HUVEC cells, and treating with 1 μM ICI 182,780 led to decrease ANGPT-2 expression in the 786-O and Caki-1 cells and phosphorylation of Tie-2 in HUVEC cells (Fig. 6e).

Importantly, treating with 1 μM ICI 182,780 can also suppress the oeANGPT-2-increased angiogenesis using endothelial HUVEC cell tube formation (Fig. 6f, h) and adding oeANGPT-2 increased HUVEC resistance to the sunitinib treatment (Fig. 6g, i).

Finally, we found that treating HUVEC cells with 1 μM Tie-2 inhibitor (Cayman chemical, #948557-43-5,) also decreased the phosphorylation of Tie-2 (Fig. 6e, right), as well as decreased the oeANGPT-2-induced angiogenesis using endothelial tube formation in HUVEC (Fig. 6j) and decreased the oeANGPT-2-increased sunitinib resistance of HUVEC cells (Fig. 6k).

Together, results from (Fig. 6a–k) suggest that targeting the ERβ/ANGPT-2/Tie-2 signaling with the ICI 182,780 can alter the sunitinib resistance via altering the HUVEC cell tube formation.

### Preclinical studies using in vivo mouse model to prove that adding FDA-approved anti-estrogen ICI 182,780/Faslodex can increase the sunitinib sensitivity to better suppress RCC progression

**Fig. 7:**
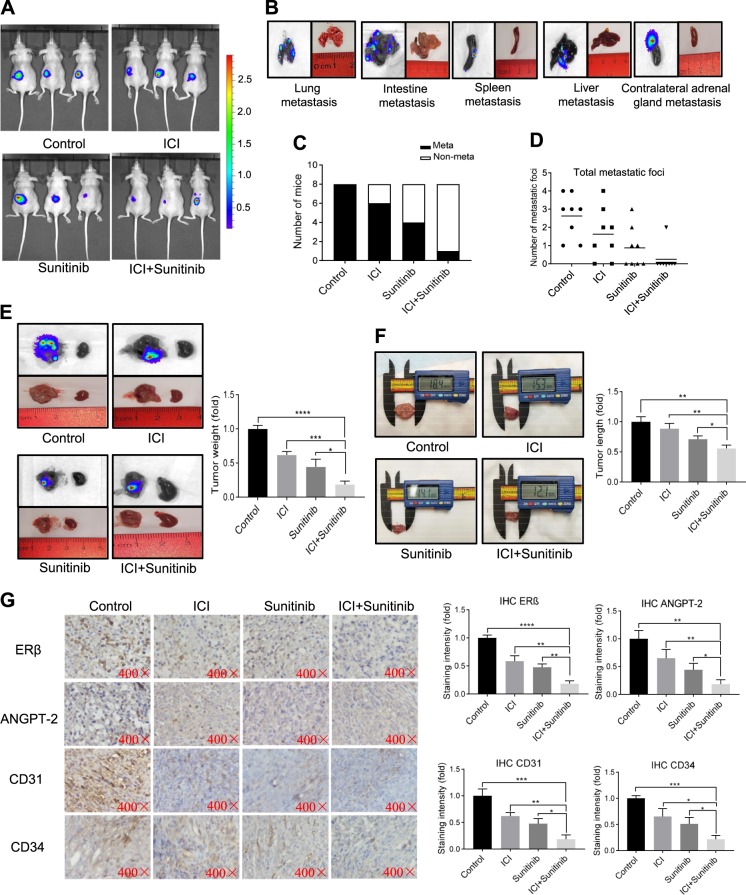
Preclinical studies using in vivo mouse model to prove that treating with FDA-approved anti-estrogen ICI 182,780 (ICI, Faslodex) can increase the sunitinib sensitivity to better suppress RCC progression. **a** IVIS imaging of four treatment groups: (1) control; (2) ICI 182,780; (3) Sunitinib; and (4) ICI 182,780+Sunitinib were used to detect various distal metastasis foci in mice with orthotopically xenografted RCC tumors under the renal capsule of the left kidney. Three representative mouse IVIS bioluminescent images are shown. **b** The IVIS images of organ bioluminescent showing the tumor metastasis to lung, intestine, spleen, liver, and the contralateral adrenal glands. **c** Quantification of the metastases in the mice. **d** Numbers of the total metastasis foci in each group of mice. **e**, **f** The mice were sacrificed and the primary tumor weights and primary tumor lengths in each group were measured. **g** Representative images of IHC results for ERβ, ANGPT-2, CD31 and CD34 in four groups of orthotopic renal tumor tissues (400×). For **e**–**g** quantitation is at the right of the images. Data are presented as mean ± SD, *P<0.05; **P < 0.01; ***P < 0.001, ****P < 0.0001 compared with the controls.

To further confirm all above in vitro cell lines data in vivo, we applied the preclinical study using the in vivo mouse RCC model with orthotopically xenografted 786-O cells expressing firefly luciferase. Thirty-two 6–8 weeks old female mice were divided into 4 groups (8 mice/group) for orthotopic left kidney implantation of 1 × 10^6^ 786-O-Luc cells. After 14 days’ tumor development, 4 groups were treated with: (1) DMSO control, (2) 0.1 mg/kg ICI 182,780, (3) 20 mg/kg sunitinib, or (4) 0.1 mg/kg ICI 182,780 + 20 mg/kg sunitinib. After 6 weeks, we found an increase of metastatic luciferase signal in the control groups (Fig. 7a). Then, we sacrificed the mice and counted the number of metastatic foci in lung, intestine, spleen, liver and contralateral adrenal gland with IVIS detection (Fig. 7b). The results revealed that combining the anti-estrogen ICI 182,780 and sunitinib led to better decrease of the total metastatic foci (Fig. 7c, d), tumor weights (Fig. 7e), and tumor lengths (Fig. 7f) than the other three groups.

IHC staining from these ccRCC xenograft tumors also demonstrated that combining the ICI 182,780 and sunitinib resulted in better decreases of the expressions of ERβ, ANGPT-2, CD31 related angiogenesis marker^37^, and CD34 related VM marker^38^ (Fig. 7g), which is consistent with our in vitro findings.

Together, results from our preclinical study using the in vivo mouse model in Fig. 7a–g prove that combining the FDA-approved anti-estrogen ICI 182,780 and sunitinib can increase the sunitinib sensitivity to better suppress RCC progression via altering the ERβ/ANGPT-2/Tie-2 pathway signaling.

## Discussion

It is well validated that the function of some oncogenes is context-dependent, such that they have different roles in different tissues, including promoting tumor growth and inhibiting tumor growth. The ERβ is a member of the nuclear receptor super-family and has been found to play important roles in invasion and metastasis of hormone-related cancers. In RCC, the ERβ promotes ccRCC migration and invasion. Angiogenesis is an essential part of the development of tumors and it is a crucial process for the progression and metastasis of RCC, and anti-vascular drugs are used in RCC patients^39^. Sunitinib, as a multi-target inhibitor of tyrosine kinases, was used to effectively suppress tumor angiogenesis and RCC growth, resulting in increase of progression-free survival in patients. Accordingly, our study attempted to examine the role of ERβ in RCC patient survival and sunitinib efficacy through a more physiological setting of endothelial cells co-culture with RCC cells, to see whether the phenotypes of endothelial cells have changed in this co-culture microenvironment.

The cancer microenvironment, including the extracellular matrix (ECM), endothelial cells and immune cells, constitute an important factor of tumor growth and invasion^40^. The interaction between tumor cells and endothelial cells through the intricate network of cytokines, seems to be critical^41^. The Angiopoietin/Tie-2 signaling system is very important for vascular angiogenesis. ANGPT-2 is a secreted glycoprotein and plays a key role in angiogenesis in cancer neovascularization. Tie-2 is a new receptor tyrosine kinases that is important in angiogenesis, particularly for vascular network formation in endothelial cells^42^. Some research shows that ANGPT-2 is both an agonist and an antagonist of Tie-2, such that ANGPT-2 can act as either a weak Tie-2 agonist or a dose-dependent Tie-2 antagonist^43^. Hu et al.^44^ reported that through Tie-2-independent pathways involving integrin-mediated signaling, ANGPT-2 stimulates tumor angiogenesis, invasion, and metastasis. In our research, we demonstrated that in RCC cells, ERβ, through transcriptional regulation, directly regulates the downstream gene ANGPT-2. Also, under the co-culture system, the RCC cells secreted large amounts of ANGPT-2 that could affect phosphorylation of Tie-2 in endothelial cells, thereby causing the angiogenesis of endothelial cells.

The antiestrogen, ICI 182,780, is a steroidal molecule that can inhibit E2-mediated activity and induce apoptosis of cancer cells related to estrogen-dependency^45^. It was demonstrated that in ovarian cancer cells, E2 treatment could enhance the c-myc expression and blocking estrogen signaling may also suppress cancer progression^46^. In bladder cancer (BCa), the combination of Bacillus Calmette Guerin (BCG) and the anti-estrogen ICI 182,780 led to better suppression of BCa than BCG alone^47^. In renal cancer, ICI 182,780 can block the ERβ/TGF-β1/SMAD3 signals pathway, thereby better inhibiting the progression of RCC^14^. Also, we used ICI 182,780 treatment in two different renal cancer cells to verify the inhibition of the angiogenesis of endothelial cells caused by renal cancer, and increased sensitivity to sunitinib. Interestingly, we targeted the ERβ/ANGPT-2/Tie-2 signaling-mediated angiogenesis with ICI 182,780 to increase the sunitinib sensitivity to better suppress RCC metastasis.

**Fig. 8:**
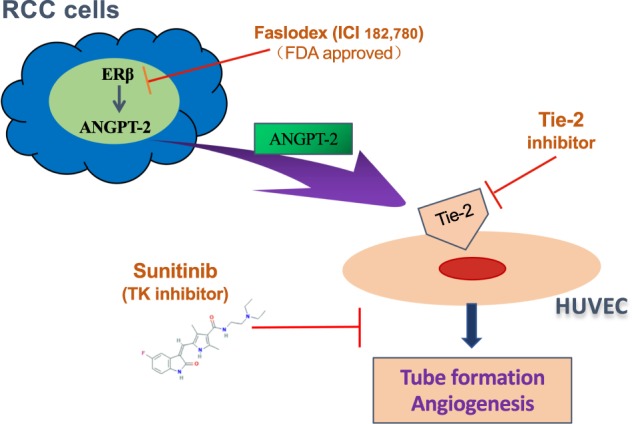
ERβ up-regulated ANGPT-2 in RCC cells can function via a paracrine mechanism to promote the endothelial tube formation (angiogenesis). The cartoon shows the regulatory mechanism how the RCC ERβ can up-regulate the expression of ANGPT-2. The increased ANGPT-2 can be secreted by RCC cells to bind to the Tie-2, an ANGPT-2 receptor specifically expressed on endothelial cells. Targeting the ERβ/Angiopoietin-2/Tie-2 signaling-mediated angiogenesis with the antiestrogen ICI 182,780 (Faslodex) or Tie-2 inhibitor may help in the development of a novel combined therapy with sunitinib to better suppress the RCC progression.

In summary, ERβ could promote ccRCC progression via altering the ANGPT-2/Tie-2 signaling-mediated angiogenesis and inhibiting the ERβ/ANGPT-2/Tie-2 signals with antiestrogen ICI182,780 may increase the sunitinib sensitivity to better suppress ccRCC progression (Fig. 8).

## Supplementary information

Supplementary Table S1
