# Supplementary figures and images for: Targeting the ERβ/Angiopoietin-2/Tie-2 signaling-mediated angiogenesis with the FDA-approved anti-estrogen Faslodex to increase the Sunitinib sensitivity in RCC
Source: Cell Death Dis. 2020 May 14;11(5):367. doi: 10.1038/s41419-020-2486-0 (PMC7224303; doi:10.1038/s41419-020-2486-0)

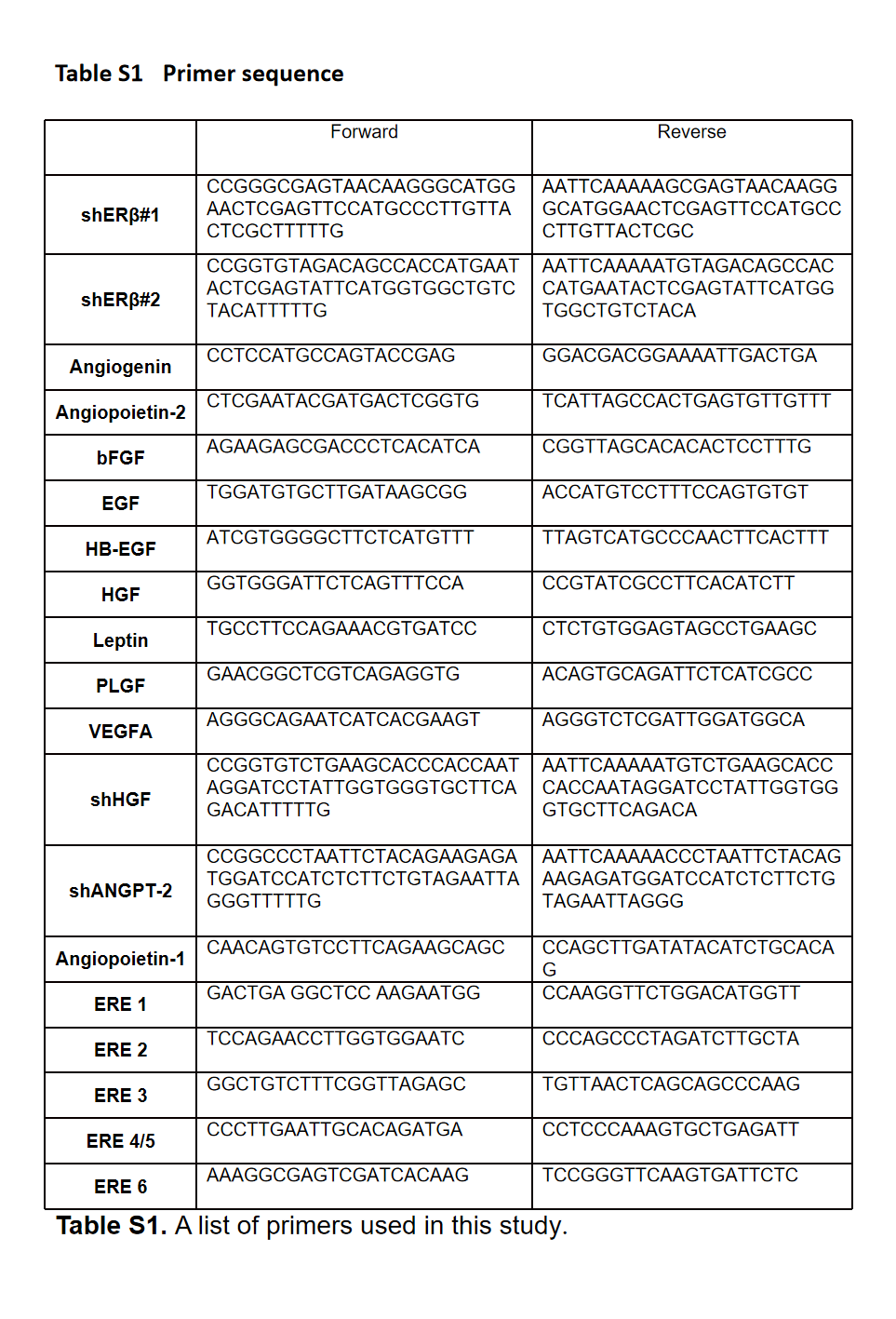

Supplement: Supplementary file 1 — Supplementary Table S1 [file 41419_2020_2486_MOESM1_ESM.tif]
